# Gender differences in the comorbidity of neurological and psychological disorders in a large clinical sample of children

**DOI:** 10.1192/bjo.2021.53

**Published:** 2021-05-04

**Authors:** Valerie Brandt, Elisa Napoleone, Praveetha Patalay

**Affiliations:** Center for Innovation in Mental Health, Department of Psychology, University of Southampton, Southampton, UK; Child Outcomes Research Consortium (CORC), Anna Freud National Centre for Children and Families, London, UK; Centre for Longitudinal Studies and MRC Unit for Lifelong Health and Ageing, University College London, London, UK

**Keywords:** Neurological disorder, comorbidity, psychological disorder, multimorbidity, gender

## Abstract

This study aimed to establish rates and gender patterns of 25 comorbidities in 1912 children (72% male) with a neurological disorder and a comparison group (*n* = 40 718, 45% male) from a large clinical records data-set in child mental health services in the UK with clinician-recorded data on neurological and psychological conditions. Obsessive–compulsive disorder, oppositional defiant/conduct disorders, autism spectrum disorders and intellectual disabilities (also known in UK health services as learning disabilities) occurred significantly more often in both boys and girls with neurological disorders than in the comparison group. Girls with neurological disorders showed a ‘male-typic’ comorbidity profile.

Neurological disorders and neurodevelopmental disorders have been shown to be associated with some psychological disorders (as defined in chapter 5, ICD-10) in youths.^1^ At least 30% of adults in primary care have also been diagnosed with a mental health condition^2,3^ and studies point to a link between neurological conditions and some psychological conditions in childhood, such as epilepsy and attention–deficit hyperactivity disorder (ADHD).^4^ Even though it is well-known that many neurological and psychological disorders have a gender bias, for example ADHD, autism spectrum disorder (ASD), conduct disorder, tic disorders and Parkinson disease are more commonly diagnosed in males, whereas anxiety disorders, mood disorders and multiple sclerosis are more commonly diagnosed in females,^5^ little is known about gendered comorbidity patterns between neurological and mental conditions. This study investigates the co-occurrence of a large number of psychological symptoms in children with a neurological disorder in a clinical sample from child mental health services. We also establish gender patterns of psychological comorbidities in children and adolescents with neurological disorders.

## Method

### Participants

The data are from the clinical records of statutory and voluntary child and adolescent mental health services taking part in the Child and Young People's Improving Access to Psychological Therapies (CYP IAPT) service transformation programme in England. The clinical records were collected April 2011–October 2015. Greater detail about these data are reported elsewhere.^6^ In this period, data on *n* = 122 923 patients were recorded; *n* = 43 156 included data on the Current View tool,^7^ where clinicians indicate the patients’ presenting problems (e.g. ADHD symptoms).

Participants between 3 and 18 years were included in this study (excluding *n* = 526: 366 under 3 years, 116 over 18 years old and 44 with missing age), resulting in an analysis sample of *n* = 42 630 (46.2% male), mean age of those with a neurological disorder 11.02 years (s.d. = 3.38); comparison group  mean age 12.41 (s.d. = 3.52).

### Measures

#### Clinician-reported presenting problems

Clinicians indicated the child's presenting problems on the Current View tool,^7^ which consists of a list of psychological diagnostic categories (e.g. depression; see the supplementary material for full list available at https://doi.org/10.1192/bjo.2021.53) rated on a severity scale (none, mild, moderate, severe). It also comprises a list of 14 further neurological and developmental disorders such as ‘PDD [pervasive developmental disorder]/autism/Asperger's’, ‘learning disabilities’ and ‘neurological disorders’ (e.g. tics/Tourette, cerebral palsy, speech and language disorders, e.g. apraxia of speech; common causes: brain injury, tumour) for which clinicians indicate if they are present or not (yes/no).

To ensure the quality of the data, clinicians are provided with detailed guidance on how to complete this tool, which includes definitions and descriptions for each item and descriptors of the response options.^7^ Details of the list of conditions and examples, as provided on the tool, are available in the Supplement material. Based on clinicians’ assessments using this tool, 1912 records were identified as being for individuals with a neurological disorder (72% male). The remaining 40 718 records (45% male) constitute the clinical comparison group in this study.

### Analysis

The prevalence of 25 different types of presenting problems in children with a neurological disorder is compared with prevalence rates in the clinical comparison group (overall and by gender, χ^2^-tests).

### Ethics statement

The data used in this study are patient records and specific consent or ethical permission was not required to conduct this analysis. All data management and confidentiality protocols governing the use of the data-set were followed. The manuscript was prepared according to the STROBE guidelines.

## Results

Clinicians reported no other presenting problems in 23% of individuals with neurological disorders, with 77% demonstrated symptoms of at least one psychological disorder.

**Fig. 1:**
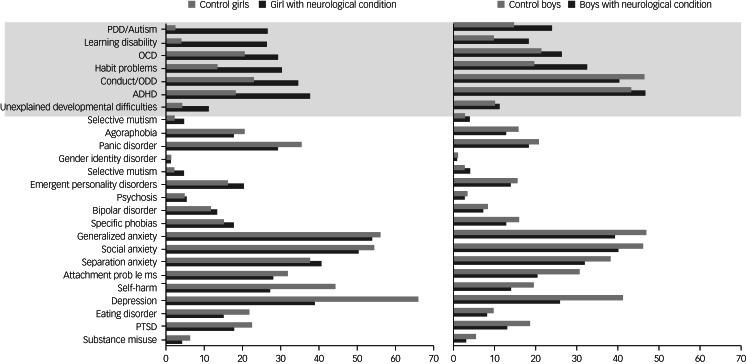
Rates of psychological disorders in the neurological disorder group and comparison group separately by gender. The findings suggest that the eight symptom domains in the grey shaded area are specifically comorbid with neurological disorders. The remaining disorders are coexisting disorders and occur at increased rates in the clinical comparison sample. PDD, pervasive developmental disorder; OCD, obsessive–compulsive disorder; ODD, oppositional defiant disorder; ADHD, attention–deficit hyperactivity disorder; PTSD, post-traumatic stress disorder.

Symptoms of ADHD, conduct disorder/oppositional defiant disorder (ODD), habit problems, obsessive–compulsive disorder (OCD), intellectual disability (also known as learning disability in UK health services), ASD and unexplained developmental disorders were significantly more common in individuals with neurological disorders than the comparison group (Fig. 1, which includes details by gender, and Supplementary Table 1). Symptoms of depression, anxiety, self-harm, substance misuse, attachment problems and eating disorder were less frequent in individuals with neurological disorders compared with the comparison group.

The disorders with the highest co-occurrence with neurological disorders were intellectual disabilities (14.5%), followed by ASD (12.8%).

## Discussion

This is the first study to compare gender patterns in mental health symptoms in a large sample of children with neurological disorders to unrelated children of the same age with at least one psychological presenting problem specified by a trained clinician.

Overall, symptoms of ADHD, OCD/habit problems, ASD and intellectual disability were more common in children with neurological disorders than in the comparison group, whereas depression and anxiety symptoms were less common. The results are partly in line with a previous study, showing an association between neurodevelopmental disorders and ADHD/behaviour problems.^1^ There was a slight difference in mean age between the groups with and without neurological disorder. This could, at least partially explain differences in comorbidities with a later onset, such as depression, self-harm, agoraphobia and substance misuse.

### Gender patterns

A unique contribution of this study is the investigation of gender patterns in neurological–mental disorder comorbidities, which has received little attention to date. Girls with neurological disorders showed a similar comorbidity pattern to boys with neurological disorders (even though the typical gender differences in comorbidities can be found within the neurological disorders group), with girls with neurological disorders displaying a male-typical comorbidity pattern (increased occurrence of ADHD, ODD, ASD, intellectual disability; decreased occurrence of depression, self-harm and eating disorders). This raises the question of whether this set of disorders have similar aetiology.

### Strengths and limitations

The main strengths of this study are the use of a large, detailed clinical data-set of patient records, permitting much more detailed analyses of patterns and outcomes of comorbid symptoms of neurological disorders in children than has been previously possible.

The use of clinician-reported disorders rather than formal diagnosis is a limitation of the study. Furthermore, the scale in the IAPT only makes it possible to check whether any neurological disorder was present or absent, without further details of what type of neurological disorder, barring further detailed analysis of these conditions. Given the scale of the data-set and it being collected as part of routine clinical care, the Current View tool aimed to record conditions based on expert clinical judgement, without the burden of diagnostic interviews across all conditions. This approach is likely to have led to higher recorded prevalence across all disorders in our study. However, the similar levels of comorbidities found in this study as previous investigations in clinical samples ^8,9^ provides support for the utility and validity of the approach taken. The setting of the study, mental health services data, precludes conclusions being extended to the co-occurrence of these conditions in the general population. Owing to the relatively low prevalence of some of these conditions, very large population-based studies would be necessary to examine this in the general population. Lastly, given the use of health records we cannot differentiate between gender at birth and self-identified gender as there might be variation in what is recorded by services and we used this variable as available.

### Implications

Psychiatric conditions of a neurodevelopmental nature such as ADHD, ASD and intellectual disability are more likely to be comorbid in children with neurological conditions; this is especially stark in females where these conditions are all less likely to occur; highlighting the possibility of shared aetiology between these conditions.

## Data Availability

Data was collected by the Child Outcomes Research Consortium (CORC) and is available at CORC upon written request.

## Supplementary material

For supplementary material accompanying this paper visit http://dx.doi.org/10.1192/bjo.2021.53. click here to view supplementary material
