# Supplementary material for: Gender differences in the comorbidity of neurological and psychological disorders in a large clinical sample of children
Source: BJPsych Open. 2021 May 4;7(3):e96. doi: 10.1192/bjo.2021.53 (PMC8142543; doi:10.1192/bjo.2021.53)
Supplement: Supplementary file 1 [file S2056472421000533sup001.docx]

| *Supplementary Table 1.* Comorbidity prevalences in children with and without neurological disorders and sex-related comorbidity prevalences. The first three columns show % prevalence of comorbidity in children with neurological disorders versus those in the comparison group and whether they occur significantly more often in one of the groups. The following 6 columns show the same for girls and boys separately. | | | | | | | | | |
| --- | --- | --- | --- | --- | --- | --- | --- | --- | --- |
| Comorbidity % | Neurological disorder  (N =1912) | Comparison group  (N = 40718) | Chi^2^ | Girls neurological disorder  (N = 534) | Girls  Comparison group  (N = 22333) | Chi^2^ | Boys neurological disorder  (N = 1373) | Boys  Comparison group  (N = 18339) | Chi^2^ |
| ADHD | 44.3 | 29.5 | **189.72** | 37.8 | 18.2 | **132.77** | 46.8 | 43.3 | 6.54 |
| Conduct/ODD | 38.7 | 33.6 | **21.04** | 34.6 | 23.0 | **39.91** | 40.3 | 46.6 | **20.59** |
| Habit problems | 32.1 | 16.3 | **318.97** | 30.3 | 13.5 | **122.60** | 32.7 | 19.8 | **130.36** |
| OCD | 27.2 | 20.8 | **45.42** | 29.4 | 20.6 | **24.71** | 26.4 | 21.5 | **18.64** |
| Learning disability | 20.5 | 6.7 | **518.61** | 26.4 | 4.0 | **608.05** | 18.3 | 9.9 | **95.37** |
| PDD / Autism | 24.7 | 9.1 | **505.21** | 26.6 | 2.5 | **1004.28** | 24.0 | 14.8 | **82.48** |
| Unexplained developmental difficulties | 11.2 | 6.9 | **50.81** | 11.2 | 4.3 | **58.77** | 11.3 | 10.2 | 1.71 |
| Depression | 29.7 | 54.9 | **468.11** | 39.0 | 66.1 | **169.55** | 26.0 | 41.3 | **124.49** |
| Self-harm | 17.8 | 33.3 | **200.06** | 27.2 | 44.5 | **63.53** | 14.1 | 19.7 | **25.36** |
| Attachment problems | 22.6 | 31.4 | **65.94** | 27.9 | 31.9 | 3.84 | 20.5 | 30.8 | **63.42** |
| Separation anxiety | 34.4 | 38.0 | **10.19** | 40.6 | 37.8 | 1.75 | 32.0 | 38.3 | **21.88** |
| Social anxiety | 43.0 | 50.8 | **43.7** | 50.4 | 54.5 | 3.65 | 40.2 | 46.2 | **18.38** |
| Generalized anxiety | 43.4 | 52.0 | **55.07** | 53.9 | 56.2 | 1.06 | 39.3 | 47.0 | **30.64** |
| Panic disorder | 21.3 | 29.0 | **52.36** | 29.4 | 35.6 | 8.87 | 18.3 | 20.9 | 5.38 |
| Agoraphobia | 14.3 | 18.4 | **21.1** | 17.8 | 20.6 | 2.46 | 12.9 | 15.9 | 8.51 |
| Specific phobias | 14.9 | 14.4 | 0.29 | 17.8 | 15.2 | 2.73 | 13.8 | 13.5 | 0.09 |
| Bipolar disorder | 9.0 | 10.3 | 3.03 | 13.5 | 11.8 | 1.48 | 7.4 | 8.5 | 2.09 |
| Psychosis | 3.6 | 4.2 | 1.81 | 5.4 | 4.8 | 0.50 | 2.8 | 3.5 | 1.5 |
| Substance abuse | 3.3 | 6.0 | **22.74** | 4.1 | 6.3 | 4.10 | 3.1 | 5.6 | **16.03** |
| PTSD | 14.4 | 20.9 | **46.02** | 17.8 | 22.6 | 6.87 | 13.1 | 18.7 | **26.99** |
| Eating disorder | 10.0 | 16.4 | **54.66** | 15.0 | 21.8 | **14.31** | 8.2 | 9.8 | 4.13 |
| Emerging personality disorder | 15.8 | 16.0 | 0.05 | 20.4 | 16.2 | 6.83 | 14.0 | 15.7 | 3 |
| Selective mutism | 4.3 | 2.5 | **27.78** | 4.7 | 2.2 | **14.42** | 4.2 | 2.9 | 7.71 |
| Gender identity disorder | 1.0 | 1.3 | 1.2 | 1.3 | 1.4 | 0.01 | 0.9 | 1.1 | 0.7 |
| Elimination problems | 7.8 | 5.1 | **27.04** | 8.4 | 3.2 | **42.89** | 7.6 | 7.3 | 0.05 |

PDD = pervasive developmental disorder; ADHD = attention deficit hyperactivity disorder, OCD = obsessive-compulsive disorder, ODD = oppositional defiant disorder;

The term “Gender identity disorder” is used in the ICD-10 as a diagnosis. It will be outdated with the publication of ICD-11, but is the term used in the mental health service dataset that was used in this analysis.

Chi square results in bold are Bonferroni-corrected, i.e. *p* < .002

**List of psychological disorders included in the Current View Tool**

| Separation anxiety (Anxious away from caregivers) |
| --- |
| Social anxiety/phobia (Anxious in social situations) |
| Generalized anxiety (Anxious generally) |
| Obsessive compulsive disorder; OCD (Compelled to do or think things) |
| Panic disorder (Panics) |
| Agoraphobia (Avoids going out) |
| Specific phobia (Avoids specific things) |
| Habit problems (Repetitive problematic behaviours) |
| Depression (Depression/low mood) |
| Self-injury or self-harm (self-harm) |
| Bipolar disorder (Extremes of mood) |
| Psychosis (Delusional beliefs and hallucinations) |
| Substance abuse (Drug and alcohol difficulties) |
| Attention deficit hyperactivity disorder; ADHD (Difficulties sitting still or concentrating) |
| Conduct disorder; CD or oppositional defiant disorder; ODD (Behavioural difficulties) |
| Post-traumatic stress disorder; PTSD (Disturbed by traumatic event) |
| Anorexia/Bulimia (Eating issues) |
| Attachment problems (problems in attachment to parent/ carer) |
| Emerging personality disorder (Persistent difficulties managing relationships with others) |
| Unexplained developmental difficulties |
| Learning disability |
| Autism/Asperger's (Pervasive Developmental Disorders)  Selective mutism (Does not speak)  Gender identity disorder (gender discomfort issues)  Elimination problems (doesn’t get to toilet in time) |
